# Investigation of Laboratory and Clinical Features of Primary Dysmenorrhea: Comparison of Magnesium and Oral Contraceptives in Treatment

**DOI:** 10.7759/cureus.32028

**Published:** 2022-11-29

**Authors:** Soner Gök, Berfin Gök

**Affiliations:** 1 Obstetrics and Gynecology, Pamukkale University, Denizli, TUR; 2 Obstetrics and Gynecology, Denizli State Hospital, Denizli, TUR

**Keywords:** vitamin d, visual analogue scale, primary dysmenorrhea, magnesium citrate, combination oral contraceptive

## Abstract

Background

The goal of this study was to identify the demographic, clinical, and laboratory characteristics of primary dysmenorrhea (PD) patients, as well as to compare the treatment options of 200 mg magnesium citrate (MgS) and combination oral contraceptive (COC) therapy.

Methods

This is a case-controlled prospective study consisting of 172 women with PD and a control group consisting of age-matched 172 without PD. The cases in the primary dysmenorrhea group were randomly divided into two groups, with 86 patients in the first group receiving 200 mg MgS, and 86 patients in the second group receiving COC treatment. The Visual Analogue Scale (VAS) was used to measure the severity of dysmenorrhea. VAS scoring was performed on the control group subjects included in the study at their first evaluation and the PD group subjects before treatment and at the third month of treatment (after three menstrual cycles).

Results

When compared to healthy controls, the PD patients had significantly more menstrual bleeding (p = 0.005), more history of maternal dysmenorrhea (p < 0.001), lower serum calcium (p < 0.001), lower serum 25-hydroxyvitamin D3 (p < 0.001) and more 25-hydroxyvitamin D deficiency (p < 0.001). When compared to the MgS group, the patients in the COC group had significantly lower VAS scores and less need for painkillers following treatment (p < 0.001). The MgS treatment group had significantly lower post-treatment VAS values than pre-treatment VAS values.

Conclusion

Lower serum calcium and 25-hydroxyvitamin D levels were found in the presence of PD. In addition, it was observed that the administration of 200 mg MgS to PD patients significantly reduced pelvic pain in dysmenorrhea, although not as much as COC administration, and caused significant reductions in the need for painkillers.

## Introduction

Dysmenorrhea is a type of pelvic pain associated with menstruation [1]. Dysmenorrhea is divided into two categories: primary and secondary dysmenorrhea [2]. Primary dysmenorrhea (PD) is the most common cause of menstrual pain that occurs without any underlying pathology [1]. Although the exact cause of this condition is unknown, many causes are linked to pain [3]. The level of prostaglandin and/or vasopressin hormones in the endometrial fluid increases, causing dysrhythmic uterine contractions and arterioles vasoconstriction. This reduces blood flow to the uterus [4].

PD, which affects 50% to 90% of women and is characterized by persistent cramping and pelvic pain, is the most common reason for consulting a gynecologist [5]. Studies conducted in North America, China, Australia, Turkey, and Iran have shown that the incidence of PD in women varies with society [6]. It has been reported that the pain associated with PD reduces women's quality of life [7] and makes it difficult for them to participate in daily activities [8,9]. When looking at the prevalence of dysmenorrhea in Turkey, it is worth noting that the prevalence ranges from 34% to 89.6% [10].

The diagnosis of PD is based on the clinical history and physical evaluation. Generally, a thorough history and physical examination are sufficient, while laparoscopy may be necessary for diagnosis. Transvaginal ultrasound may be used in cases where secondary dysmenorrhea is suspected [4]. PD usually begins six to 24 months after menarche. Pelvic pain that occurs before or shortly after menarche may be associated with obstructive genital tract malformations and warrant further evaluation [11].

Nonsteroidal anti-inflammatory drugs (NSAIDs) are the first-line treatment for dysmenorrhoea pain relief in women who do not want contraception, and treatment with one of the traditional NSAIDs at a therapeutic dose should be attempted for at least three menstrual cycles [12,13]. Hormonal contraceptives are an effective way to treat primary dysmenorrhea when an NSAID has failed, is contraindicated, or contraception is needed. The most commonly studied hormonal agent, estrogen-progestin oral contraceptives, inhibits the proliferation of the endometrial lining [5]. Thus, it reduces the production of prostaglandins in the uterus. It is effective in the treatment of dysmenorrhea in randomized clinical trials [5,11]. Magnesium’s muscle relaxant and vasodilator properties also prevent prostaglandin synthesis. Studies have reported that a daily intake of 250 mg of magnesium is effective for dysmenorrhea [14].

Primary dysmenorrhea is a common women's health problem that adversely affects the work life, school life and psychology of especially young women. Clinical and laboratory features of PD may differ according to age, ethnicity and geographic region. Magnesium citrate is a smooth muscle relaxant in addition to its analgesic properties and has very few side effects. Therefore, this study aimed to determine the demographic, clinical, and laboratory characteristics of PD, and to compare 200 mg magnesium citrate (MgS) and low-dose combination oral contraceptive (COC) (3 mg drospirenone and 0.02 mg ethinylestradiol) treatment on reducing pain severity. First, serum calcium, magnesium, 25-hydroxy vitamin D, and vitamin B12 levels were evaluated in the study and compared to a control group of healthy volunteers without PD. 

## Materials and methods

This case-controlled prospective study was conducted in Pamukkale University gynecology clinic between November 2019 and November 2020 in accordance with the principles of the Declaration of Helsinki. All participants provided written and informed consent prior to participating in the study. Ethics committee approval was obtained from the Pamukkale University Clinical Research Ethics Committee (08.10.2019: 17).

Study participants and design

A total of 393 female university students between the ages of 18 and 25 who had been consecutively admitted to the study center’s department of gynecology were eligible for the study. Eighteen women who had pelvic pathologies, 10 women who had systemic diseases, nine women who had acute infections, five women who regularly used calcium or vitamin D supplements, and seven women who refused to participate were excluded. A total of 172 women were randomly allocated to the primary dysmenorrhea group if they had regular menstrual cycles (21-35 days, with menstruation lasting three to seven days) and had at least four consecutive painful periods in the previous six months, with pain beginning one day before or on the day of onset of bleeding. Cases with severe PD, that is, with a visual analog scale (VAS) score of more than six, were included in the PD group of our study. The control group consisted of 172 women who did not have dysmenorrhea (primary and secondary). Randomization was accomplished using opaque envelopes that were sequentially numbered and sealed. Individuals with a history of metabolic, autoimmune, neurological, or psychiatric diseases or cancer, those using an intrauterine device, with a history of abdominal and/or pelvic surgery, and presenting findings pointing to secondary dysmenorrhea were excluded from the study.

The participants were asked to fill out a guided self-assessment questionnaire that included demographic information and clinical characteristics related to menstruation. All participants' age, marital status, weight, height, menarche age, menstrual cycle length, menstrual cycle duration, amount of menstrual bleeding, presence of history of maternal dysmenorrhea were questioned and recorded. The BMI was calculated as follows: Body mass index (BMI) = Body weight (kg)/Body height2 (m2). The severity of primary dysmenorrhea pain was determined using a VAS scoring system, which is based on a numerical rating between 1 and 10.

Patients diagnosed with PD were randomly allocated to either the MgS group or COC group using the papers with the words “MgS” and “COC” which were put in an envelope, and the paper each participant drew out determined the group. Patients in the first group (n = 86) were administered 200 mg MgS. The patients in the second group (n = 86) were administered low-dose COC treatment. There was no blinding in the study. The patients were informed in detail about the effects and side effects of the drugs they used. Both groups received treatment for three menstrual cycles. Two women who did not attend the control examination and three women who did not comply with the treatment were excluded from the MgS group. One woman who did not attend the control examination, two women who did not comply with the treatment, and three women who discontinued the treatment due to side effects were excluded from the COC group. After treatment, 81 cases from the MgS group and 80 cases from the COC group were included in the evaluation.

Study plan and interventions

In the study, MgS was administered at 200 mg per day during menstruation, starting on the first day of the premenstrual phase when signs of menstruation appeared. The low-dose COC (3 mg drospirenone and 0.02 mg ethinylestradiol) (24/4, Yazz, Bayer, Germany) treatment group, on the other hand, started on the first day of menstruation and continued for the duration of the cycle. Both groups of patients received treatment for three menstrual cycles. The participants in both groups were informed that they could use painkillers when the need for painkillers occurred during the two menstrual periods from the beginning of the treatment, but they should not use painkillers since the VAS score would be re-measured in the third menstruation. The patients were re-evaluated on the fourth and fifth days of the third cycle, and their pain scores were re-done. It was recorded whether or not they needed additional painkillers.

Measurements

Venous blood samples were taken from all subjects included in the study after 12 hours of fasting, on 3-5 days of the cycles. Calcium (Ca), magnesium (Mg), vitamin B12, and 25-hydroxy vitamin D levels in serum from the samples were studied.

Pelvic ultrasonographic imaging was performed in all cases by the same investigator on the same day that serum samples were taken.

The VAS was used to measure the severity of dysmenorrhea. According to the VAS, "no pain" is usually graded as 0 points, and "worst pain imaginable" as 10 points (10 cm scale). Pain intensity ranges from 3 to 6, with 3 being mild, 3-6 being moderate, and > 6 being severe. VAS scoring was performed on the control group subjects included in the study at their first evaluation and the PD group subjects before treatment and at the third month of treatment (after three cycles).

Statistical analysis

Statistical analysis was conducted using SPSS version 25.0 software (IBM Corp., Armonk, NY, USA). Numerical variables are expressed in mean and standard deviation and categorical variables in numbers and percentages (%). Numerical data for normal distributions were analyzed by Skewness. Independent sample t-tests were used to analyze differences between the groups. The significance threshold was chosen at p < 0.05. 

## Results

Table 1 compares the demographic, clinical and biochemical characteristics of the primary dysmenorrhea and control groups. There was no significant difference in demographic characteristics such as age, marital status, weight, height, and BMI between the PD and control groups (p = 0.879, 0.750, 0.152, 0.165, and 0.463, respectively). When compared to healthy controls, the dysmenorrhea patients had significantly more menstrual bleeding (p = 0.005), history of maternal dysmenorrhea (p < 0.001), lower serum calcium (p < 0.001), lower serum 25-hydroxyvitamin D3 (p < 0.001), 25-hydroxyvitamin D deficiency (p < 0.001), and higher VAS scores (p < 0.001). There were no differences in menarche, menstrual cycle length, menstrual cycle duration, serum magnesium, or serum vitamin B12 levels between the two groups (p = 0.086, 0.211, 0.078, 0.931, and 0.931, respectively).

**Table 1:** Demographic, clinical and biochemical characteristics of the participants * p < 0.05 is considered statistically significant; PD: primary dysmenorrhea; VAS: visual analog scale

Variable	Women with PD (n=172)	Healthy control (n=172)	p*
Age (year)	21.17±2.22	21.21±2.20	0.879
Marital status			
Single	136 (%85)	138 (%86.3)	0.750 (χ²=0.102)
Married	24 (%15)	22 (%13.8)	
Weight (kg)	58.60±6,8	59.67±6.50	0.152
Height (m)	1.64±0.05	1.65±0.04	0.165
BMI (kg/m^2^)	21.73±2.50	21.92±2.25	0.463
Menarche (age)	12.29±0.90	12.11±0.91	0.086
Menstrual cycle length (days)	27.43±1.88	27.16±1.96	0.211
Menstrual cycle duration (days)	5.23±1.12	5.01±1.09	0.078
Menstrual bleeding (pads/day)	6.25±1.22	5.83±1.40	0.005*
History of maternal dysmenorrhea			
Yes	69 (%43.1)	33 (%20.6)	<0,001* (χ²=18.651)
No	91 (%56.9)	127 (%79.4)	
Serum calcium (mg/dl)	8.42±0.29	8.85±0.25	<0,001*
Serum magnesium (mg/dl)	2.20±0.19	2.20±0.19	0.931
25-hydroxyvitamin D (ng/ml)	21.70±6.41	43.28±10.65	<0,001*
25-hydroxyvitamin D deficiency	130 (%81.3)	21 (%13.1)	<0,001* (χ²=148.98)
Kobalamin (B12) (pg/ml)	308.87±69.23	309.52±65.20	0.931
Visual analog scale score	7.68±1.15	1.59±0.64	<0,001*

Table 2 compares the demographic, clinical, and biochemical characteristics of the treatment groups. Demographic and clinical characteristics and laboratory results were not different between the two groups (p > 0.05). When compared to the MgS group, the patients in the COC group had significantly lower VAS scores and less need for painkillers following treatment (p < 0.001).

**Table 2:** Demographic, clinical and biochemical characteristics of the treatment groups * p < 0.05 is considered statistically significant; MgS: magnesium citrate; COC: combination oral contraceptive; VAS: visual analog scale

Variable	MgS group (n=81)	COC group (n=80)	p*
Age (year)	21.05±2.23	21.30±2.20	0.477
Marital status			
Single	68 (%85)	68 (%85)	1.000 (χ²=0.000)
Married	12 (%15)	12 (%15)	
Weight (kg)	59.09±6.28	58.11±7.25	0.365
Height (m)	1.64±0.05	1.65±0.05	0.104
BMI (kg/m^2^)	22.09±2.49	21.37±2.46	0.067
Menarche (age)	12.25±0.89	12.34±0.91	0.541
Menstrual cycle length (days)	27.48±1.90	27.40±1.85	0.801
Menstrual cycle duration (days)	5.23±1.10	5.24±1.13	0.944
Menstrual bleeding (pads/day)	6.23±1.21	6.28±1.23	0.796
Hystory of maternal dysmenorrhea			
Yes	36 (%45)	33 (%41.3)	0.632 (χ²=0.229)
No	44 (%55)	47 (%58.7)	
Serum calcium (mg/dl)	8.44±0.29	8.40±0.27	0.366
Serum magnesium (mg/dl)	2.21±0.19	2.20±0.19	0.746
25-hydroxyvitamin D (ng/ml)	21.80±6.43	21.61±6.42	0.854
25-hydroxyvitamin D deficiency	64 (%80)	66 (%82.5)	0.685 (χ²=0.164)
Kobalamin (B12) (pg/ml)	297.80±80.64	319.95±53.79	0.043*
VAS score (before treatment)	7.68±1.15	7.69±1.14	0.945
VAS score (after treatment)	5.14±2.42	3.15±1.79	<0,001*
Need for painkillers (after treatment)	54 (%67.5)	26 (%32.5)	<0,001*

Table 3 compares the VAS values before and after treatment in the PD treatment groups. The MgS treatment group had significantly lower post-treatment VAS values than pre-treatment VAS values (p < 0.001*). Similarly, the COC treatment group had significantly lower post-treatment VAS values than pre-treatment VAS values (p < 0.001).

**Table 3:** VAS values before and after treatment in the PD group * p < 0.05 is considered statistically significant; PD: primary dysmenorrhea; MgS: magnesium citrate; COC: combination oral contraceptive; VAS: visual analog scale

Variable	VAS (before treatment)	VAS (after treatment)	p*
MgS Group (n=80)	7.68±1.15	5.14±2.42	<0,001*
COC group (n=80)	7.69±1.14	3.15±1.79	<0,001*

## Discussion

In the current study, we aimed to investigate the levels of vitamin D, calcium, magnesium and vitamin B12 in order to contribute to the elucidation of the etiology of primary dysmenorrhea and to test the idea of comparing 200 mg MgS and COC treatment in the treatment of primary dysmenorrhea. The main finding was that the primary dysmenorrhea group had more menstrual bleeding and lower serum calcium and 25-hydroxyvitamin D3 levels compared to the control group. In addition, we found that although the decrease in VAS scores of patients treated with 200 mg MgS among primary dysmenorrhea patients did not decrease as much as those treated with COC, they decreased statistically significantly and reduced the need for additional painkillers.

Obesity may play a role in the etiology of PD. A significant relationship between PD and anthropometric measurements such as height, waist, and hip circumferences has been discovered in research conducted with women with PD [15,16]. Contrary to these results, studies have also reported that there is no significant relationship between PD and anthropometric measurements (body weight, BMI) and body compositions (body fat ratio, waist-hip ratio) [17]. In the study by Rafique et al. [18], the prevalence of PD was found to be significantly higher in underweight (BMI < 18.5) female students aged 18-25 years compared to overweight students (BMI > 30 kg/cm3). Akunna et al. [19] determined in their study that the severity of dysmenorrhea rose as the BMI of university students increased. In the current study, when the PD group was compared to the control group, no statistically significant difference was found in weight, height, or BMI measurements. Different results have been reported regarding the relationship between the presence of PD and BMI. We hypothesize that this may be due to factors such as geographic location, age, dietary habits, and acquired habits in stress management among the participants.

The PD group had more menstrual bleeding, according to Karacin et al. [20]. In our current study, when PD cases were compared with control group cases, there was no significant difference in menstrual cycle length and menstrual cycle duration, but we found that PD cases had more menstrual bleeding. We believe that this increase in menstrual bleeding may be due to the stress caused by pain in dysmenorrhea cases, which may lead to an increase in blood bradykinin and prostaglandin levels, resulting in dilatation of the pelvic blood vessels and an increase in flow.

Vitamin D is a biologically inert molecule that is activated by hydroxylation, first to 25-hydroxyvitamin D3 by 25α-hydroxylase in the liver, and then to 1,25-dihydroxyvitamin D3 by 1α-hydroxylase in the kidney. This activation process induces intestinal absorption of calcium and phosphate. When serum vitamin D levels decrease, intestinal calcium absorption is reduced significantly. As a result, calcium levels in the extracellular fluid decrease [21]. The female reproductive system relies heavily on 25-hydroxyvitamin D. Vitamin D receptors have been found in ovarian and endometrial tissue and epithelial cells of the fallopian tubes, decidua, and placenta [22]. Some studies have reported a link between 25-hydroxyvitamin D deficiency and early dysmenorrhea, due to the regulatory effect of calciferol on prostaglandin levels [23]. A link was found between low calcium intake or 25-hydroxyvitamin D deficiency and dysmenorrhea in adolescent and young women [22]. Karacin et al. [20] concluded that calcium and 25-hydroxyvitamin D levels were lower in the presence of PD, and 25-hydroxyvitamin D deficiency was higher when compared to the control group. In our current study, we found that serum calcium and 25-hydroxyvitamin D levels were lower and the rate of 25-hydroxyvitamin D deficiency was higher in the presence of PD. Vitamin D has been reported to reduce prostaglandin production. Therefore, we are of the opinion that vitamin D deficiency contributes to the development of PD in two different ways, first by causing an increase in prostaglandin production, which causes pelvic pain, and secondly, by decreasing calcium absorption, causing uterine muscle spasms and contractions due to hypocalcemia.

Treatment options for PD include analgesic drugs, oral contraceptives, prostaglandin synthetase inhibitors, dietary changes, and other psychiatric treatment approaches. In many studies, COC has been shown to be effective in the treatment of PD [5,11]. When NSAIDs are unsuccessful, contraindicated, or there is a desire for contraception, COC is an effective treatment option. However, their long-term use may be challenging due to undesirable side effects. In our study, VAS scores of the COC group decreased significantly after the treatment, which was consistent with the literature. Some studies reported that decreased magnesium levels were observed in patients with PD [24]. Magnesium has been suggested as a potential treatment for PD [25]. The effect of magnesium on PD has been attributed to calcium channel antagonist activity or inhibition of prostaglandin F2 biosynthesis, though the precision of these processes has yet to be confirmed [26]. Shin et al. [27] reported that the optimal magnesium dose for the treatment or prevention of dysmenorrhea is uncertain. In our study, there was no significant difference in serum Mg levels between the PD and control groups. However, a significant decrease was observed in VAS scores after 200 mg MgS treatment in PD cases, as well as a significant decrease in the requirement for additional painkillers after treatment. These results support the thesis that 200 mg MgS, which has almost no side effects, may be a good alternative in reducing pelvic pain, especially in young women with PD who do not want to use too many analgesics or COCs because of their undesirable side effects. 

The following were the limitations of the current study: (1) it was a single-centre study; (2) treatment outcomes were evaluated only after three menstrual cycles and (3) only 200 mg dose of MgS was administered.

## Conclusions

Lower serum calcium and 25-hydroxyvitamin D levels were found in the presence of PD. These findings are consistent with the literature. In addition, it was observed that the administration of 200 mg MgS to PD patients significantly reduced pelvic pain, although not as much as COC administration, and caused significant reductions in the need for painkillers. It has been demonstrated that MgS, which has almost no side effects, can be used safely in the treatment of generally young PD patients. More research is needed to clarify the role of vitamin D in the pathogenesis of primary dysmenorrhea and prove the efficacy of magnesium treatment.
